# Structure and Functions of Gap Junctions and Their Constituent Connexins in the Mammalian CNS

**DOI:** 10.1134/s1990747821020069

**Published:** 2021-06-10

**Authors:** E. Yu. Kirichenko, S. N. Skatchkov, A. M. Ermakov

**Affiliations:** a Academy of Biology and Biotechnology, Southern Federal University, Rostov-on-Don, 344090 Russia; b Department of Biochemistry, School of Medicine, P.O. Box 60327, Universidad Central del Caribe, Bayamón, PR, 00960-6032 USA; c Department of Physiology, School of Medicine, P.O. Box 60327, Universidad Central del Caribe, Bayamón, PR, 00960-6032 USA; d Faculty of Bioengineering and Veterinary Medicine, Don State Technical University, Rostov-on-Don, 344003 Russia

**Keywords:** gap junctions, connexins, astrocytes, neurons

## Abstract

Numerous data obtained in the last 20 years indicate that all parts of the mature central nervous system, from the retina and olfactory bulb to the spinal cord and brain, contain cells connected by gap junctions (GJs). The morphological basis of the GJs is a group of joined membrane hemichannels called connexons, the subunit of each connexon is the protein connexin. In the central nervous system, connexins show specificity and certain types of them are expressed either in neurons or in glial cells. Connexins and GJs of neurons, combining certain types of inhibitory hippocampal and neocortical neuronal ensembles, provide synchronization of local impulse and rhythmic activity, thalamocortical conduction, control of excitatory connections, which reflects their important role in the processes of perception, concentration of attention and consolidation of memory, both on the cellular and at the system level. Connexins of glial cells are ubiquitously expressed in the brain, and the GJs formed by them provide molecular signaling and metabolic cooperation and play a certain role in the processes of neuronal migration during brain development, myelination, tissue homeostasis, and apoptosis. At the same time, mutations in the genes of glial connexins, as well as a deficiency of these proteins, are associated with such diseases as congenital neuropathies, hearing loss, skin diseases, and brain tumors. This review summarizes the existing data of numerous molecular, electrophysiological, pharmacological, and morphological studies aimed at progress in the study of the physiological and pathophysiological significance of glial and neuronal connexins and GJs for the central nervous system.

## INTRODUCTION

Gap junction, or nexus, is the conductive type of cell-to-cell contact providing direct transition of small water-soluble molecules with a molecular weight of no more than 1.5 kDa (inorganic ions, sugars, amino acids, nucleotides, vitamins, etc.) from the cytoplasm of one cell into the cytoplasm of another cell. Their existence was noted for the first time in the study of the ultrastructure of neurons in the neural circuit of craw-fish in 1953 [1]. A little later, these structures were described as single five-layer plates tightly connecting cellular membranes and supposedly involved in the electrical conductivity of cardiomyocytes [2]. The first evidence of the presence of GJs in the mammalian CNS was obtained in the 1960s–1970s [3–6]. Then their functional significance was intensively studied, with electrophysiological recording of currents from the pairs of adjacent neurons, injection of fluorescent dyes and assessment of their intercellular distribution, followed by ultrastructural studies of their localization in different parts of the CNS. Numerous data of the past 30 years demonstrate that all regions of the central and peripheral nervous systems, from the brain, retina and olfactory bulb to the spinal cord, ganglions and enteroglia, contain cells connected by GJs [7–9]. GJ subunits (connexin proteins) are generally assembled into hexameric complexes, or membrane hemichannels (connexons). By puncturing the membrane lipid bilayer of two contacting cells, connexons form a hexagonal pore of 1.5 nm in diameter. When two contacting cells assemble one GJ from two connexons, the diversity of the connexon subunits – connexins – provides specificity and multiplicity of functions in neurons [10], astrocytes [11–13], and satellite and enteric glia [14, 15]. Connexons are assembled in such a way that adjacent membranes are separated by a gap of 2–4 nm in width (hence the term “gap junction”). Electron microscopy makes it possible to visualize GJ connexons on replicas as hundreds of densely grouped circular twisted rosettes forming peculiar plaques [16, 17].

The CNS cells connected by GJs can be both glial and neuronal. Glial GJs are formed mainly between macroglial cells, capable of accumulating polyamines, and provide metabolic coupling of the cells [18. 19]. Neuronal GJs provide electrotonic cooperation between the cells and function as electrical synapses [20, 21]. Thus far there is no distinct morphological confirmation of the existence of GJs between neurons and glial cells. In parallel with the studies described above, GJ structure, channels, proteins comprising these channels, cytoplasmic and extracellular domains, as well as their degradation, were studied to determine the potential mechanisms for regulation of their functional state and intercellular conduction. The present review is aimed at describing the data on peculiar features of the molecular structure of GJs, the connexin proteins comprising them, as well as their functions in CNS, available in worldwide scientific literature.

## CHARACTERISTICS OF THE GENES OF CONNEXINS AND THEIR DOMAIN STRUCTURE

According to the modern nomenclature available in the Entrez Gene Global Search System of Whole Genomes, the connexin genes are divided into 5 groups: alpha, beta, gamma, delta and epsilon. In each group, new genes were numbered as they were described; for example, *GJA1* is the first alpha-group connexin described in humans. This gene encodes a connexin of 43 kDa (Cx43). At present, the connexin family comprises about 20 individual proteins differing from each other in the molecular mass and in the range of tissue expression [22, 23]. The CNS of mammals was shown to contain connexin 36 (Cx36), connexin 45 (Cx45), connexin 43 (Cx43), connexin 30 (Cx30), connexin 26 (Cx26), connexin 32 (Cx32), etc. (Table 1).

Traditionally, connexin is designated as Cx for animals and as CX for humans; genes of animal and human connexins are designated as *Gj* and *GJ*, respectively. The initial concepts of domain structure of all connexin-encoding genes were rather simple. They consisted of one exon with the 5′-terminal untranslated region and the second exon with the main coding region and the 3′-terminal untranslated region [24]. Further detailed study of the structure of these genes showed that their coding regions could be interrupted by noncoding introns, e.g., in the *Gjd2* gene encoding Cx36 [25], the *Gjd4* gene encoding Cx39 [26], and the *Gja10* gene encoding Cx57 [27].

Subsequent molecular studies demonstrated the existence of several splice isoform transcripts in one connexin gene. By way of illustration, the connexin 32 gene proved to have two different splice variant transcripts in rats and humans [28, 29] and three splice variants in cows and mice [30, 31]. Moreover, it was shown that most of these isoforms emerged due to the 5′-regions and the alternative use of promoter regions; the coding region of the gene in mRNA was unchanged. The expression of such transcript isoforms is specific to different tissues and organs. In hepatocytes and secretory acinar cells of the pancreas, the Cx32 mRNA transcription begins at the promoter upstream the first exon; in the Schwann cells of CNS, it begins at the so-called neuron-specific promoter located between the first and second coding exons [32]. The existence of such different variants of tissue-specific transcripts in mammals has been shown for many connexins (Cx40, Cx43, Cx45, Cx30, etc.), which is believed to be due to peculiar features of the family of these genes. In the classical conception, the expression of connexins is largely regulated at the level of transcription with the involvement of different transcriptional factors and epigenetic modifications such as histone transformations or DNA methylation [33]. However, the relevant literature also describes the regulatory factors for expression at the level of translation, e.g., the IRES (internal ribosome entry site) or uORFs (upstream open reading frames) elements in the connexin genes [32].

## CONNEXIN SYNTHESIS AND MOLECULAR STRUCTURE

**Fig. 1.**
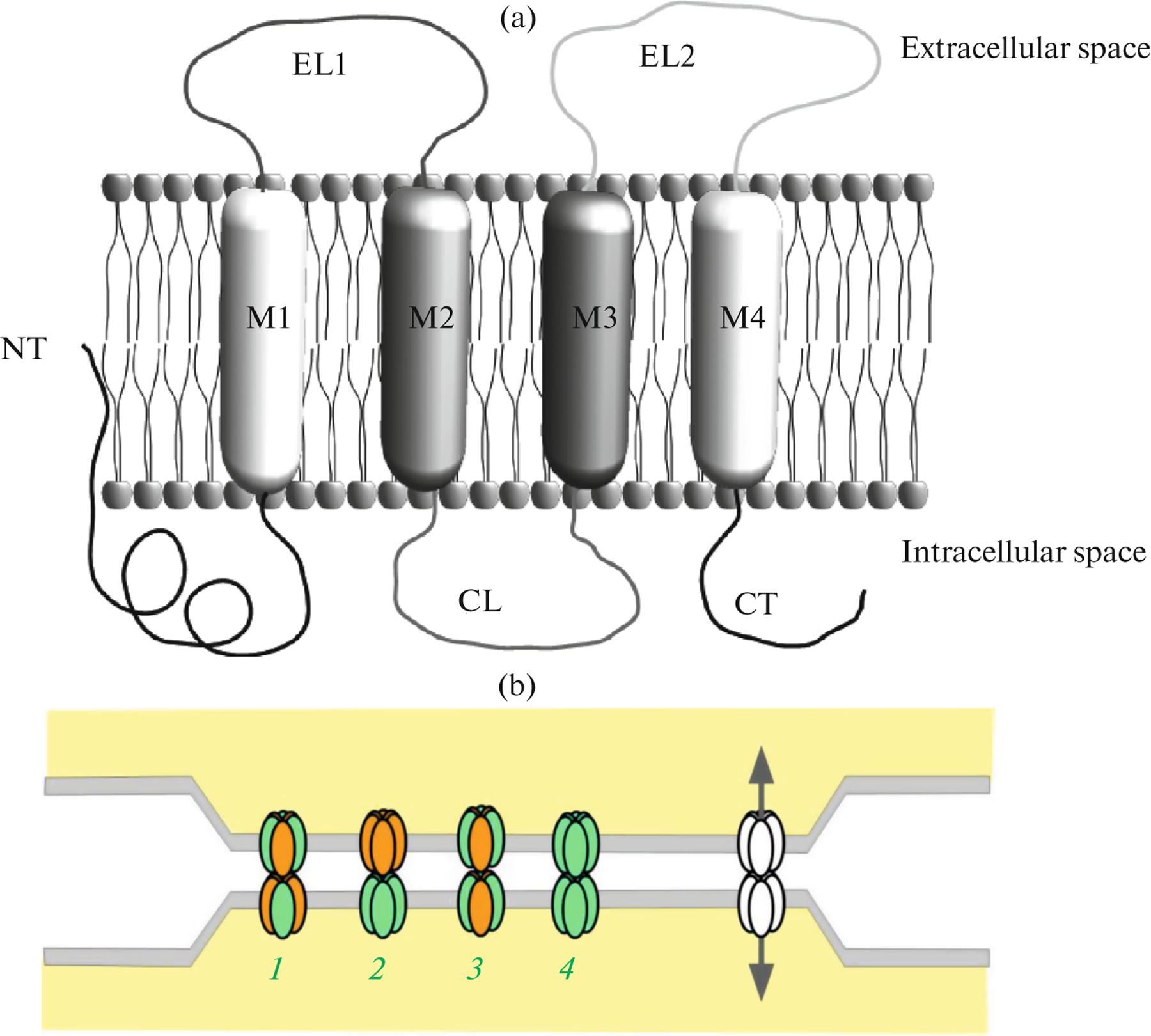
Schematic representation of a connexin molecule and a gap junction. (a) The domain structure of one connexin molecule embedded into the membrane lipid bilayer. Designations: M1–M4, transmembrane domains; EL1–EL2, extracellular loops; CL, intracellular loop; NT, N terminus of the molecule; CT, C terminus. (b) Aggregation of individual channels, from 10 to several thousands, results in the formation of a cluster, or a plaque, of gap junctions. The distance between the plaques (transmembrane “gap”) is about 2–3 nm. Each connexon is composed of six connexin molecules (subunits). Types of connexons of the gap junctions: *1*, heteromeric heterotypic; *2*, homomeric heterotypic; *3*, heteromeric homotypic; *4*, homomeric homotypic.

The synthesis of connexins is a continuous dynamic process due to their short half-life and the necessity of permanent replacement. Like other membrane proteins, connexins are synthesized on the endoplasmic reticulum (ER) membrane with subsequent oligomerization, transport through the Golgi apparatus, and embedding into the cell membrane [34]. Each connexin molecule as an unstable monomer consists of four hydrophobic transmembrane helical domains (M1–4), two extracellular loops (extracellular loop 1 (EL1) and extracellular loop 2 (EL2)), one cytoplasmic loop (CL), and free N-, C-terminal domains of the molecule also designated as NT and CT (T is for tail) (Fig. 1a).

Oligomerization, or the assembly of connexins into hexameric complexes, proceeds differently depending on the type of connexin. On the basis of structural homology, connexins can be divided into two separate groups with respect to the process of oligomerization. Connexins encoded by the *Gjb1–Gjb7* genes (the so-called β-connexins, including Cx26 and Cx32) take a more conventional pathway, where complete oligomerization into hexameric hemichannels is necessary before the transport from ER to the cis-Golgi apparatus [35]. Other connexins (non-β-connexins Cx43, Cx40, Cx46) are stabilized by a connexin-specific monomer and transported to the trans-Golgi network for oligomerization at the late stages of the secretory pathway [36]. After oligomerization, connexins acquire their original structure as a protein complex of 6 connexin molecules – a connexon, and are delivered to the plasma membrane for incorporation and assembly into GJ plaques [37]. Crystallographic analysis of a hemichannel has shown that connexons have a positively charged cytoplasmic entrance, a funnel, a negatively charged transmembrane region, and an extracellular cavity. In the funnel, there is a narrowing formed by six N-terminal helices lining the channel wall. Such structure limits the size of molecules passing through the channel [38].

The cytoplasmic and extracellular domains of membrane-embedded connexin molecule perform different functions. For instance, the extracellular loops of the molecule are involved in making links to connexins of adjacent cells, while the NT- and CT-ends of the molecule are responsible for the small molecule selectivity of the channel [37]. The length of cytoplasmic loops, like the length of C-domains, varies between connexins. In addition, there are so-called phosphorylation sites at the C-end [39]. Phosphorylation of these C-terminal regions of connexins by kinases is one of the methods of posttranslational modification of these proteins. Posttranslational modification of connexins, in turn, regulates many important aspects of their life cycle, including synthesis, transport, channel gating, and protein–protein interactions [40]. Modern data suggest that the promising mechanism of influencing the GJs and hemichannels can be precisely the effect on particular kinases, which allows regulation of the life span of connexins, their migration to the plasma membrane, the assembly of hemichannel plaques, GJ formation and pore permeability [41]. In addition, the interaction between the C-end and quite a number of kinases determines its interaction with other proteins and thereby modulates its signaling function.

## DEGRADATION OF CONNEXINS AND GAP JUNCTIONS

The half-life of connexins is about 1.5 h, and the causes of such a short protein lifespan are yet unclear [42]. GJ hemichannels undergo both proteasomal and lysosomal degradation with the preceding internalization via combined endo/exocytosis [43]. This process occurs through GJ plaque invagination into the cell cytoplasm, separation from the plasma membrane, and formation of a double-membrane annular gap junction, or “connexosome”, with its subsequent degradation [44]. At the same time, one of the cells connected by GJs is a donor of connexosome, which is taken up by another cell. The ultrastructural studies of connexosome degradation demonstrate that this process probably occurs via lysosomes or autolysosomes, since it has been noted that the five-layer structure of GJs inside these organelles becomes “fuzzy” in some places [45]. There are at least two types of such cytoplasmic vesicles involved in GJ internalization: large and slowly degrading vesicles (about 0.5–5 μm in diameter) and smaller ones degrading within a few seconds (0.18–0.27 μm in diameter) [46]. Connexins taken up thereby undergo recirculation and are transported either back to the plasma membrane or to the Golgi complex [47].

## TYPES OF CONNEXONS AND THEIR COUPLING IN GAP JUNCTIONS

During oligomerization, connexons can be composed of connexins of the same type (homomeric connexons) or connexins of different types (heteromeric connexons). Accordingly, the GJs composed by homomeric or heteromeric connexons can be homotypic or heterotypic, depending on connexon composition. The relevant literature describes different connexon combinations and GJ types: homomeric homotypic, heteromeric homotypic, homomeric heterotypic, and heteromeric heterotypic (Fig. 1b). The functional properties (permeability and selectivity for different molecules and ions) in heterotypic channels formed by two different connexins can be different from the respective properties of homotypic channels [48]. For example, heterotypic GJs and their functional difference from homotypic GJs are discussed by Lin et al. [49], who have shown that the GJs of heart ventricles contain only Cx43, while atrial GJs consist of Cx40 and Cx43. The co-expression of these connexins is the major cause of the most of dynamic gating properties of atrial GJs compared to ventricular GJs due to their different polyamine regulation. These differences in the properties of atrial and ventricular GJs can play particular roles in the genesis of slowed myocardial conduction and arrhytmias [49].

The mechanisms of connexin compatibility in heterotypic contacts have not yet been well elucidated. Theoretically, the alignment of two connexins creates up to 196 variants of different channels. However, it has been shown that the docking of two hemichannels with the formation of a single functional GJ channel is possible only between compatible connexins; the molecular mechanism of this process is unclear. It is supposed that the mechanism of docking of hemichannels into a single GJ pore is based on hydrogen bonding, its disruption being the cause of channelopathies [50]. The most important element of connexin molecule for selective docking and formation of functional channels is considered to be extracellular domain E2 but not E1 [51]. At the same time, the docking of hemichannels of a particular pair of connexons depends on the estimated number of hydrogen bonds at each E2–E2 docking interface. The study of the crystal structure of a GJ channel showed the formation of about 36 hydrogen bonds altogether at six docked extracellular domains of E2–E2 [38, 52]. At the same time, the amino acid residues formed after the docking of E1–E1 and E2–E2 domains may contain different mutations associated with human genetic diseases such as Charcot–Marie–Tooth disease (peripheral neuropathy) [53], dysplasias, cardiac arrhythmias, congenital cataracts, deafness, etc. [54–57].

## CHARACTERISTICS AND FUNCTIONS OF CONNEXINS IN THE CNS

It is necessary to characterize the expression of different connexins in nerve tissue in order to understand the functional significance of hemichannels and GJs they create. In the CNS, the functions and properties of GJs depend on whether they connect nerve or glial cells.

### Neuronal Connexins

#### Connexin 36 (Cx36).

The Cx36 gene expression (like the expression of other connexins) is usually determined by researchers using the method of RNA-scope in situ hybridization, the *lacZ* reporter gene, and the analysis of synthesized protein by the methods of Western blot, immunohistochemistry and electron immunohistochemistry. The functions of connexin 36 are studied mainly by electrophysiological methods in knockout and transgenic animals. The GJs consisting of Cx36 are formed by only homomeric connexons [58]; the pore formed by the latter is functioning with a very low conductivity of about 10–15 pS [59]. The basic function of GJs containing Cx36 is defined by researchers as electrical coupling and synchronization of intercellular pulse activity of neurons.

During the early postnatal development, different types of already differentiated neurons are combined through GJs. This connection not only creates a network of cells with coordinated metabolism but also provides the transmission of nerve impulses under conditions when chemical synapses do not yet function. The revealed temporary electrical connections between neurons are provided largely by electrical synapses formed by Cx36 [60]. The number of GJs between neurons dramatically decreases by day 18 of postnatal development, while the development of chemical synapses approaches the adult values [61]. In the adult mammalian brain, Cx36 is a neuronal connexin specific for the GJ between GABAergic inhibitory neurons [62], less frequently between excitatory neurons [9, 61]. At the same time, the maximum number of such GJs connects the dendrites of GABAergic neurons expressing calcium-binding proteins and neuropeptides, e.g., parvalbumin. The morphological evidence of existence of such connections has been demonstrated by the studies in the hippocampus and in layer 4 of the neocortex (the barrel cortex) [63, 64]. The authors of these works developed the classification of parvalbumin-containing fast-spiking neurons with respect to the distal and proximal dendritic locations of GJs on these cells. In addition, such types of parvalbumin-containing neurons contained the thalamic vesicular glutamate transporter 2 in the cytoplasm of the soma and proximal dendrites, which is indicative of their involvement in direct thalamocortical connections. The existence of such networks can argue in favor of the fact that the Cx36-containing GJs are involved in the control of excitatory bonds, provide the regulation of thalamocortical interaction and synchronization of both local impulse activity and rhythmical activity of large and small neuronal ensembles in the hippocampus and in the neocortex at alpha-, theta-, gamma- and high-frequency oscillations [65, 66]. Some of these rhythmic oscillations are supposed to participate in the processes of perception, attention concentration and memory at both cellular and systemic levels. However, such neuronal networks combined by GJs not only have been little studied for the most of cerebral areas, but even have not been described for all layers of the cortical column of the neocortex, which undoubtedly must be the goal of future neurophysio-logical and neuromorphological studies.

#### Connexin 45 (Cx45).

There are few studies of the expression of Cx45 and GJs containing this protein. Similar to Cx36, Cx45 belongs to the group of GJ proteins of CNS neurons, because they are expressed neither in oligodendrocytes nor in astrocytes [67]. The high level of the Cx45 gene expression is observed already during embryogenesis and in the first two weeks of postnatal development in all parts of the brain. After that, its expression is confined to thalamic structures, the CA3 hippocampal region and the cerebellum [67]. Considerable coincidence of the level of expression of both connexins of the developing neurons (Cx36 and Cx45) during early postnatal development suggests that they play similar roles in this period and can contribute to the functional specialization of particular subtypes of developing neurons.

In the adult brain, Cx45 has been found in pyramidal cells, which are known to form electrical connections but not to express Cx36. Such cells have been identified in the neocortex, in the hippocampus (CA1–CA4 regions) and in the thalamus [67, 68]. In addition, Cx45 is expressed in neurons of the olfactory bulb [69] and in subpopulations of neurons of the olivocerebellar system [70]. By analogy with Cx36, Cx45 protein forms rather low-conductive GJs, which are sensitive to the changes in membrane potential and close during membrane hyperpolarization [71]. At the same time, the existence of heterotypic Cx36/Cx45 GJs has not been demonstrated up to now. It is supposed that one of the basic functions of Cx45-containing GJs is synchronization of the oscillatory activity of neurons in the gamma frequency ranges [72].

### Connexins of Glial Cells

Interglial GJs are most typical not of the cells of the immune system, which originate from the mesenchyme–microglia (macrophages in the CNS and PNS), but of macroglial cells (astrocytes, oligodendrocytes, pituicytes, tanycytes, Müller and Bergmann cells). The primary macroglia originates from the ectoderm and the stem precursor in common with nerve cells—radial glia, which gives both the types of astrocytes and other glia and the types of neurons in ontogenesis. In adult organisms, the radial glia of CNS is represented by Müller cells in the retina and the Bergmann glia in the cerebellum, while in other CNS structures it is actually reduced. Nevertheless, all cells originating from the radial glia are combined by the unique ability to accumulate polyamines and to perform intercellular exchange of small molecules through GJs containing different types of connexins [18]. The connexins of glial cells are expressed everywhere in CNS and PNS, providing metabolic, syncytial and signaling cooperation, and play a certain role in migration of both astrocytes and neurons. Mutations in the genes of glial connexins and their deficiency are associated with numerous diseases [73], one of them being autism [74].

#### Connexin 43 (Cx43).

In the developing brain, embryonic neural progenitor cells are linked to each other through Cx43-containing GJs [75]. In addition, Cx43 is expressed by radial cells of the glia, along which young neurons migrate from the ventricular zones to neocortical layers [76]. It is believed that Cx43 does not form GJs but provides the adhesion of neurons during the migration [77]. In addition, the cytoplasmic C-terminal domain of Cx43 plays the key and crucial role in this process [75]. Cx43-containing connexons form channels with a moderate conductivity; at the same time, GJs consisting of Cx43 are low-sensitive to the changes in membrane potential and close in response to membrane depolarization [71]. In the adult brain, Cx43 is the major protein comprising the GJs of astrocytes [78], which are widespread everywhere, including the cortex, the subcortical structures, the hippocampus, and other structures.

It should be noted that neuronal GJs containing Cx36 and macroglial GJs containing Cx43 have different functions in spite of similar morphological characteristics. The GJs between neurons are rare and serve for avalanche-like synchronization of electrical signals in small cell ensembles, while the GJs of ectodermal glia are scaled and required for organization of the syncytium. Astrocyte syncytium is a three-dimensional glial framework in brain structures, which performs a number of important functions such as the control of concentrations of extracellular ions and neurotransmitters, as well as realization of metabolic processes [18, 73, 79]. The necessary condition for maintaining these functions in the syncytium is the opening of GJs, which is associated with the presence of polyamines [80]. In the adult brain, the accumulation of polyamines (spermine, spermidine, putrescine, agmatine) is the distinctive feature of astrocytes but not neurons [81–84]. The unique ability of Cx43 to maintain open GJ in the syncytium was shown by comparing Cx43 and Cx40 and by demonstrating the phenomenon of polyamine blockade of the amino acid sequence (binding site) at the N-terminus of Cx40 [85]. Such specific blinding sites for the polyamine spermine were not found in Cx43 [86]. Moreover, further studies showed the sensitivity of Cx43 to the polyamines that open GJ channels, as well as the fact (which is most important for cell physiology) that polyamines eliminate the blockade of these channels by hydrogen [87] and calcium [88] cations. The elimination of cation-induced blockade by polyamines is the crucial factor for GJ functioning in the glia, because it is just acidification of the cytoplasm followed by calcium release (the so-called calcium waves in astrocytes) that is the characteristic feature of the astrocyte syncytium but not of neuronal ensembles.

The accumulation of polyamines in glial cells is necessary not only for maintaining the open state of GJs [88, 89], but also for regulating fluxes in potassium channels of the glia [90]. The known ability of astrocytes to shunt each other and to combine single cells into the common syncytium via GJs makes it possible to implement the recently discovered property of “astrocyte isopotentiality” [91]. The essence of this property of astrocytes is as follows: the membrane potential of each cell is fixed (stabilized) due to the contact with adjacent cells, so that it is possible to maintain the collective membrane potential of the glial syncytium at the level of about −90 mV, i.e., 20–30 mV below the neuronal one, which creates conditions for the entry of potassium ions into cells against the concentration gradient. The necessity of such capture of potassium ions from extracellular environment is imposed by accumulation of excess potassium in the intercellular space during spike generation in the neurons. Potassium is transported to the glial cytoplasm from the space around the neurons through the Kir4.1 (KCNJ10) potassium channels of astrocytes. It should be noted that the Kir4.1 potassium channels (KCNJ10) are the predominant channels of glial cells localized solely in their membranes [92]; moreover, many CNS and PNS disorders are associated just with the impaired function of these channels [73, 93]. At the same time, as a result of depolarization of such syncytium at increased concentrations of potassium ions in case of epilepsy, neurotrauma or ischemia [94], astrocytes loose the ability to remove excessive potassium from the intercellular space, which leads to the inactivation of neuronal activity and coma.

In addition to the above-described mechanism, the dynamic regulation of astrocyte networks combined via GJs of Cx43 is performed through Cx43 phosphorylation by protein kinases, including protein kinase C (PKC) and tyrosine kinase [95]. Cx43 phosphorylation decreases the permeability of intercellular channels and suppresses intercellular communication through GJs. The deficiency of Cx43 protein in astrocytes leads to a decrease in the number of GJs, and calcium wave propagation is impaired [96], which directly affects the activity of neurons.

In addition of astrocytes, Cx43 was also found in most of the studied human astrocytomas and in the astroglial component of neuroglial tumors. Low-grade gliomas (more than 60% of all cases) displayed strong membrane staining after immunohistochemistry, while the most of high-grade astrocytomas showed a decrease in typical membrane and cytoplasmic localization of this protein. Immunoblotting demonstrated the presence of several Cx43 isoforms both in the control samples of the cortex and in the low-grade gliomas, though the most of malignant gliomas have only Cx43 isoform corresponding to the nonphos-phorylated form. The higher content of Cx43 protein compared to the control was found in reactive astrocytes of the perifocal zone of the epileptic focus, as well as in the perifocal zones of low-grade gliomas. This fact probably indicates the existence of a certain regulatory pathway with the involvement of Cx43 and the astroglial syncytium in tumor-prone regions. Hence, it is assumed that the high expression of the connexin genes in low-grade gliomas, as well as in the peritumoral areas, can contribute to the emergence of tumor-associated rhythmic epileptiform discharges [39, 94].

#### Connexin 30 (Cx30).

In addition to Cx43, astrocyte connexins include Cx30 and Cx26 [97]. Even in the first works it was shown that Cx30 appeared in the brain during individual development much later than Cx43 [98]. The first manifestation of the Cx30 gene expression is observed in gray matter astrocytes with different regional patterns in the developing brain closer to adulthood. The characteristic feature of Cx30 proved to be its almost complete absence in white matter. The content of this protein in subcortical structures of the brain is higher than in the neocortex [99]. The observed differences in Cx30 expression can be well associated with the function of neuronal networks, which involves some local populations of astrocytes expressing this protein. As we have shown in the previous studies of the cerebral cortex and the olfactory bulb, Cx30 is concentrated mostly in fine astrocyte branches around microcapillaries and vessels. In some GJs with the typical ultrastructure, the products of immunohistochemical reaction with anti-Cx30 antibodies are localized in only one of the contacting processes. Probably, such asymmetric contacts around the vessels are formed by two different connexins of astrocytes [100].

The experiments with neurotoxic effect showed dramatic and specific changes in the level of Cx30 mRNA expression in reactive astrocytes surrounded by neurons that have undergone apoptosis. This fact indicated the direct or indirect involvement of this type of connexin in neuronal cell death [19, 97]. It was also shown that Cx30 mutations could cause the sensory loss of hearing and various skin diseases [39]. The mice with Cx30 deficiency demonstrated the inner ear pathology associated with increased apoptosis of the cells of the cochlear sensory epithelium, which resulted in the absence of the endocochlear potential and progressive hearing loss [101].

#### Connexin 26 (Cx26).

Cx26 is the third (on a par with Cx30 and Cx43) representative of the connexins of glial cells. In the early neurogenesis, the Cx26 gene is expressed in CNS in the cells of the leptomeningeal envelope, as well as in the astrocytes and neurons of the developing brain and spinal cord [19]. Though the existence of Cx36 in Cx26-containing GJs connecting neurons and astrocytes has been assumed, no morphological evidence of co-existence of Cx26 and Cx36 has been provided [102, 103]. Currently, it is firmly believed that Cx26 is a component of heterotypic GJs between astrocytes and oligodendrocytes, because its co-localization with Cx43, Cx45, Cx30 and Cx32 is often mentioned [19]. The existence of such contacts raises the question of heterogeneity of glial cells with respect to the expression of connexins, the more complex docking of connexons of heterotypic connexin combinations targeted at the interaction between neurons and panglial networks.

Hereditary mutations in the *GJB2* gene of connexin 26 are a basis of mutilation syndromes of sensorineural deafness, hearing loss and hyperkeratosis with autosomal recessive (DFNB1) and autosomal dominant (DFNA3) types of inheritance [104, 105]. These mutations have been described as missense mutations, which result in nonconservative amino acid substitution, impaired function of extracellular loop EL1 of the Cx26 molecule, and inability of the protein to form connexons and GJs. Moreover, more and more cases of combination of the above-described syndrome and the Dandy–Walker malformation are described [106] and, hence, the *GJB2* gene is supposed to be also involved in other, similar to this malformation, hereditary diseases of known and unknown etiology.

#### Connexin 32 (Cx32).

The gene of the Cx32 protein is expressed in Schwann cells (lemmocytes) localized along the axons of peripheral nerve fibers. Cx32 connects the Schwann cell body with the myelin sheath and plays the crucial role in myelination. In addition, Cx32 is found in oligodendrocytes and participates in the functional processes associated with these cells [107, 108]. The Cx32-containing GJs of oligodendrocytes are composed mainly of heterotypic connexons and have been found between oligodendrocytes, between an oligodendrocyte and an astrocyte, as well as between successive myelin layers [19]. One of the best known diseases associated with the mutation in the Cx32 gene is the Charcot–Marie–Tooth disease, or hereditary motor sensory neuropathy [53], which is manifested by Schwann cell hyperplasia and myelin-opathy: segmental demyelination or remyelination. One of the promising targeted therapies for this hereditary disease can be delivery of a vector with the “correct” *GJB1* gene for subsequent incorporation into the human genome.

#### Innexins and pannexins.

Connexins are not the only channel-forming proteins of GJ. There is, for example, a family of proteins called innexins (invertebrate connexins). The genes of innexins encode proteins in the GJs of Drosophila, *Caenorhabditis elegans*, as well as the Mollusca, Annelida and Platyhelminthes species [109]. At the same time, innexin proteins form functional GJs [110]. Pannexin proteins (Panx1, Panx2 and Panx3) were discovered by Panchin in the embryo of the actinia *Nematostella vectensis* [111]. These proteins were recognized as homologous to the GJ proteins of invertebrates [112, 113]. The molecules of innexins bear little resemblance to connexins, with the exception of two conservative cysteine residues in their extracellular loops. However, some connexin and pannexin subunits are surprisingly similar [114]. The function of pannexins in the mammalian CNS is currently unclear. It is known that there is a distinct mRNA expression of Panx1 and Panx2 in particular neurons, including pyramidal cells and interneurons of the hippocampus [113]. However, it is difficult to answer the question of the existence of electrical synapses of the vertebrate neurons comprised by pannexins. At present, it is known that pannexins play an important biological role as the components of hemichannels, favoring the release of ATP and modulating the intercellular propagation of calcium waves [115].

## CONCLUSIONS

The research on the structure, functions, lifecycle of GJs and the proteins composing them in the cells of living organisms under normal and pathological conditions has aroused interest in the past 50 years, and the amount of these studies continues to increase. It seems relevant to study these structures in the CNS, because it is considered that cells coupled by GJs can be the morphological basis of regulatory mechanisms in the brain, which is extremely important for the correct work of the neocortex, the hippocampus, the thalamic nuclei, and other brain structures.

It is known that connexins Cx36, Cx45, Cx43, Cx30, Cx26 and Cx32 are specific and most important for the CNS among the existing individual connexins. The domain structure of the connexin genes consists of two exons, and their coding regions can be interrupted by noncoding introns. Many connexins, including connexins of the CNS, are characterized by the presence of splice isoform transcripts with tissue- and organ-specific expression. The expression of connexins can be regulated both at the transcriptional and translational levels and is a permanent active dynamic process of a cell due to the short lifetime of both the protein and the GJ. The causes of short half-life of connexins and GJs in cells are still unknown. The newly synthesized molecule of each connexin has a characteristic structure with intracellular, extracellular and membrane domains, each of them performing its own specific functions. When migrating to the plasma membrane, connexins undergo oligomerization, or the assembly of molecules into a hexagonal structure–a connexon. The docking of connexons can lead to formation of different types of gap contacts, including those in the CNS: heteromeric heterotypic, homomeric heterotypic, heteromeric homotypic, and homomeric homotypic. However, the mechanism of docking and the principle of compatibility of these hemichannels have not yet been completely investigated. Homotypic and heterotypic GJs may have different gating properties. Degradation of connexins as GJ components occurs via endo/exocytosis and formation of connexomes, followed by degradation of these structures in lysosomes or autolysosomes.

The studies of GJs of the CNS can be provisionally divided into the following two subtypes: the study of neuron–neuron GJs composed of Cx36 and/or Cx45 and the study of glia–glial GJs containing the major connexins of the macroglia (Cx43, Cx30, Cx32, Cx26, etc.). In addition to connexins, in the CNS there exist alternalive channel-forming GJ proteins—pannexins, the function of which is currently not quite clear. Despite similar morphological characteristics, Cx36-containing neuronal GJs and Cx43-containing macroglial GJs are used for different functional purposes. The existence of GJs between nerve cells is related to synchronization of both the local pulse activity and the rhythmic activity of whole groups of neurons of particular types in the developing and adult brain, which are linked by these contacts. The ensembles of inhibitory neurons connected by electrical synapses have been found in the neocortex and the hippocampus but have not yet been studied in many other brain regions. Such neuronal networks can play crucial roles in the formation and consolidation of memory, in the processing of spatiotemporal, sensory, intra-organ information, in the mechanisms of perception, attention concentration, and other cognitive processes. Glia-glial GJs and connexins promote adhesion during neuronal migration along the radial glial strands even during brain development. In the adult brain, such contacts contribute to formation of glial syncytia performing quite a number of important functions, e.g., control of the concentration of extracellular ions and neurotransmitters, as well as to implementation of metabolic processes and maintenance of intracellular and extracellular homeostasis. In addition, GJs and hemichannels can directly control the activity of both individual neurons and neuronal networks by promoting the propagation of potassium and calcium waves in the glial syncytium. The necessary condition for realization of these functions in the syncytium is GJ opening, which is due to the presence of polyamines (spermine, spermidine, putrescine, agmatine). In the adult brain, the accumulation of polyamines is a unique characteristic feature of astrocytes but not neurons.

GJs and connexins are an object of study of the pathogenesis of different diseases, including widespread CNS disorders. Congenital mutations in the connexin genes lead to hereditary diseases such as hearing loss, skin diseases, neuropathies associated with demyelination, remyelination and dysplasia of cells, autism, etc. Further analysis of peculiarities of the molecular structure, physiological and behavioral functions of GJs and connexins can contribute to the development of targeted therapies for connexin-linked diseases, including those with the involvement of gene engineering. Therapeutic agents should be targeted at the recovery of damaged connexin genes, as well as stimulation of the recovery of new proteins and GJs, and provide personalized treatment of human genetic diseases.

One should note the great significance of research on the expression of connexins and the formation of GJs in the context of neurocarcinogenesis. This research trend is at the very beginning of its development. However, even the first works in this field suggest the formation of a fresh approach to individual genetic predisposition to brain tumors, as well as new opportunities of diagnosis and treatment of CNS neoplasms. Various splice isoforms of connexins, potential regulatory regions of their genes and transcripts, the domains of connexin molecules and the control of GJ functional state can be tools for the search and creation of novel therapeutic strategies based on the regulation of connexin expression and the activity of gap junctions and hemichannels in tumors of the human brain.

## Figures and Tables

**Table 1.** The major connexins of the mammalian CNS, coding genes and their localization

Type of connexin	Gene	Mammal	Chromosome	Cells of the central nervous system
Connexin 36, Cx36	*Gjd2*, Group Delta 2	*Mus musculus* (mouse)	Chromosome 2	Neurons of the central nervous system
Connexin 43, CX43	*GJA1*, Group Alpha 1	*Homo sapiens* (human)	Chromosome 6	Astrocytes, glial tumors of CNS
Connexin 43, Cx43	*Gja1*, Group Alpha 1	*Mus musculus* (mouse)	Chromosome 10	Astrocytes
Connexin 30, CX30	*GJB6*, Group Beta 6	*Homo sapiens* (human)	Chromosome 13	″
Connexin 30, Cx30	*Gjb6*, Group Beta 6	*Mus musculus* (mouse)	Chromosome 14	″
Connexin 26, CX26	*GJB2*, Group Beta 2	*Homo sapiens* (human)	Chromosome 13	Oligodendrocytes
Connexin 62, CX62	*GJA10*, Group Alpha 10	″	Chromosome 6	B-type retinal horizontal cells
Connexin 32, CX32	*GJB1*, Group Beta 1	″	Chromosome 10	Myelinated Schwann cells
Connexin 47, CX47	*GJC2*, Group Gamma 2	″	Chromosome 1	Oligodendrocytes
Connexin 31, CX31	*GJB3*, Group Beta 3	″	Chromosome 1	Dopaminergic neurons
